# Supplementary material for: Synergistic Effects of Soil-Based Irrigation and Manure Substitution for Partial Chemical Fertilizer on Potato Productivity and Profitability in Semiarid Northern China
Source: Plants (Basel). 2024 Jun 13;13(12):1636. doi: 10.3390/plants13121636 (PMC11207225; doi:10.3390/plants13121636)
Supplement: Supplementary file 1 [file plants-13-01636-s001.zip › plants-3011703-supplementary.pdf]

Soil-based irrigation combined with partial manure substitution for chemical fertilizer improves potato productivity and profitability in semiarid northern China

Lingling Jiang [1], Rong Jiang [2], Ping He [3, *], Xinpeng Xu [3], Shaohui Huang [4], Hanyou Xie [3], Xiya Wang [3], Qiying Wu [3], Xia Zhang [1], Yi Yang [1]

[1] Shandong Key Laboratory of Biophysics, Institute of Biophysics, Dezhou University, Dezhou 253023, China; jll@dzu.edu.cn (L.J.); zhangxia@dzu.edu.cn (X.Z.); yangyi@dzu.edu.cn (Y.Y.)

[2] Institute of Plant Nutrition and Resources, Beijing Academy of Agriculture and Forestry Sciences, Beijing 100097, China; Rong_Jiang@outlook.com (R.J.)

[3] Key Laboratory of Plant Nutrition and Fertilizer, Ministry of Agriculture and Rural Affairs / Institute of Agricultural Resources and Regional Planning, Chinese Academy of Agricultural Sciences (CAAS), Bei-jing 100081, China; heping02@caas.cn (P.H.); xuxinpeng@caas.cn (X.X.); xiehyys@163.com (H.X.); 18236960892@163.com (X.W.); qiyingwww@163.com (Q.W.)

[4] Hebei Fertilizer Technology Innovation Centre, Institute of Agricultural Resources and Environment, Hebei Academy of Agriculture and Forestry Sciences, Shijiazhuang 050051, China; shao-hui1988@sina.com (S.H.)*

* Correspondence: heping02@caas.cn

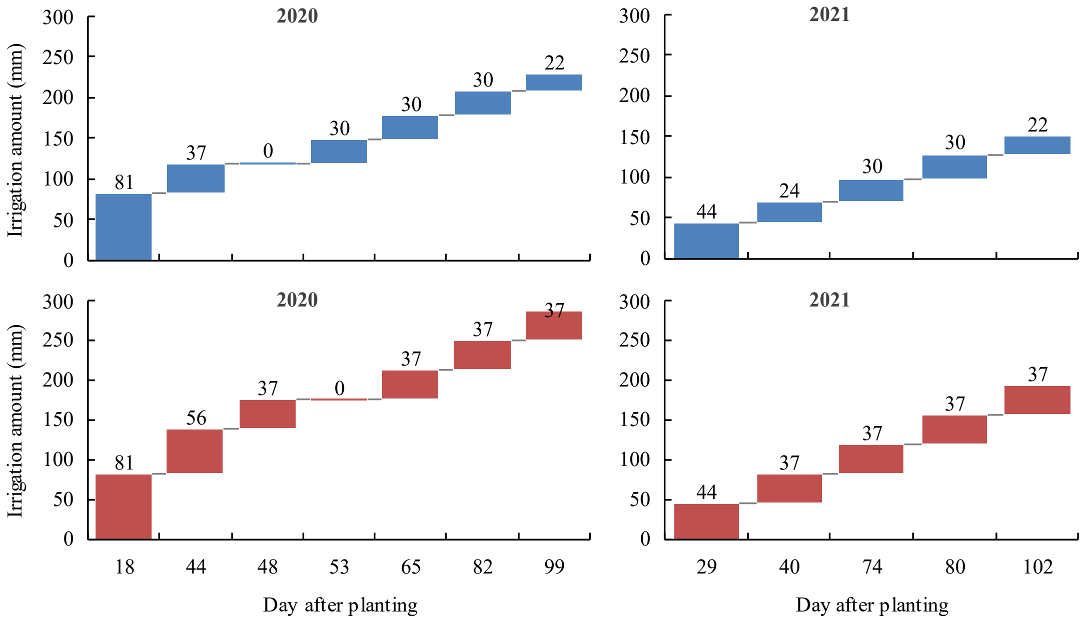

Figure S1. Irrigation timing and amount for soil-based irrigation regime (blue) and conventional irrigation regime (red) in 2020 and 2021.

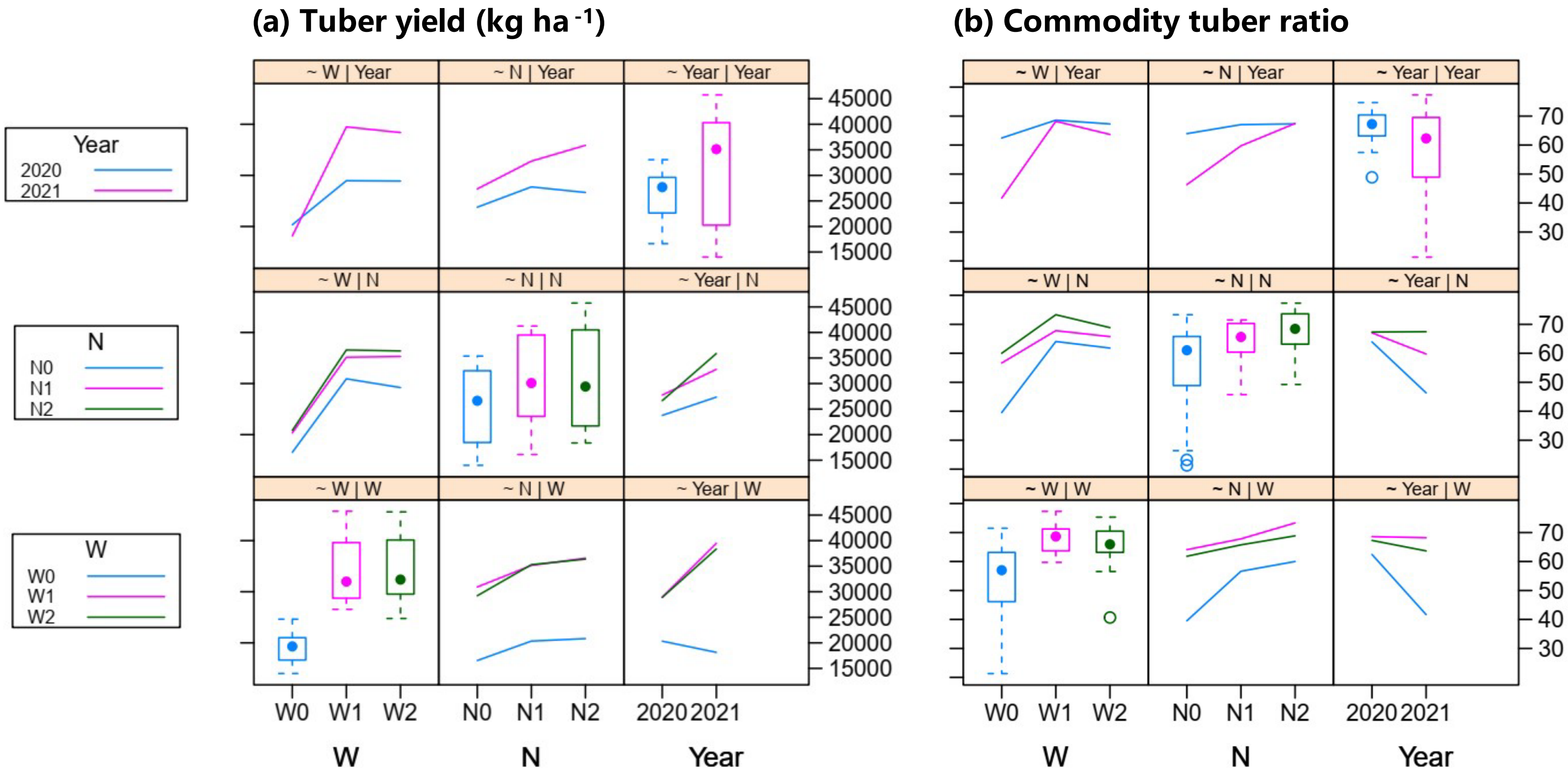

Figure S2. The main effect and 2-way interactions of water, nitrogen (N) treatments and years on the tuber yield and commodity tuber ratio of potato. Water treatments: no water irrigation (W0), soil-based water irrigation regime (W1), and farmer's conventional water irrigation regime (W2). Nitrogen treatments: no N application (N0), 100% chemical fertilizer N at 210 kg N ha^{-1} application rate (N1), and 25% substitution of chemical N with manure N (N2).

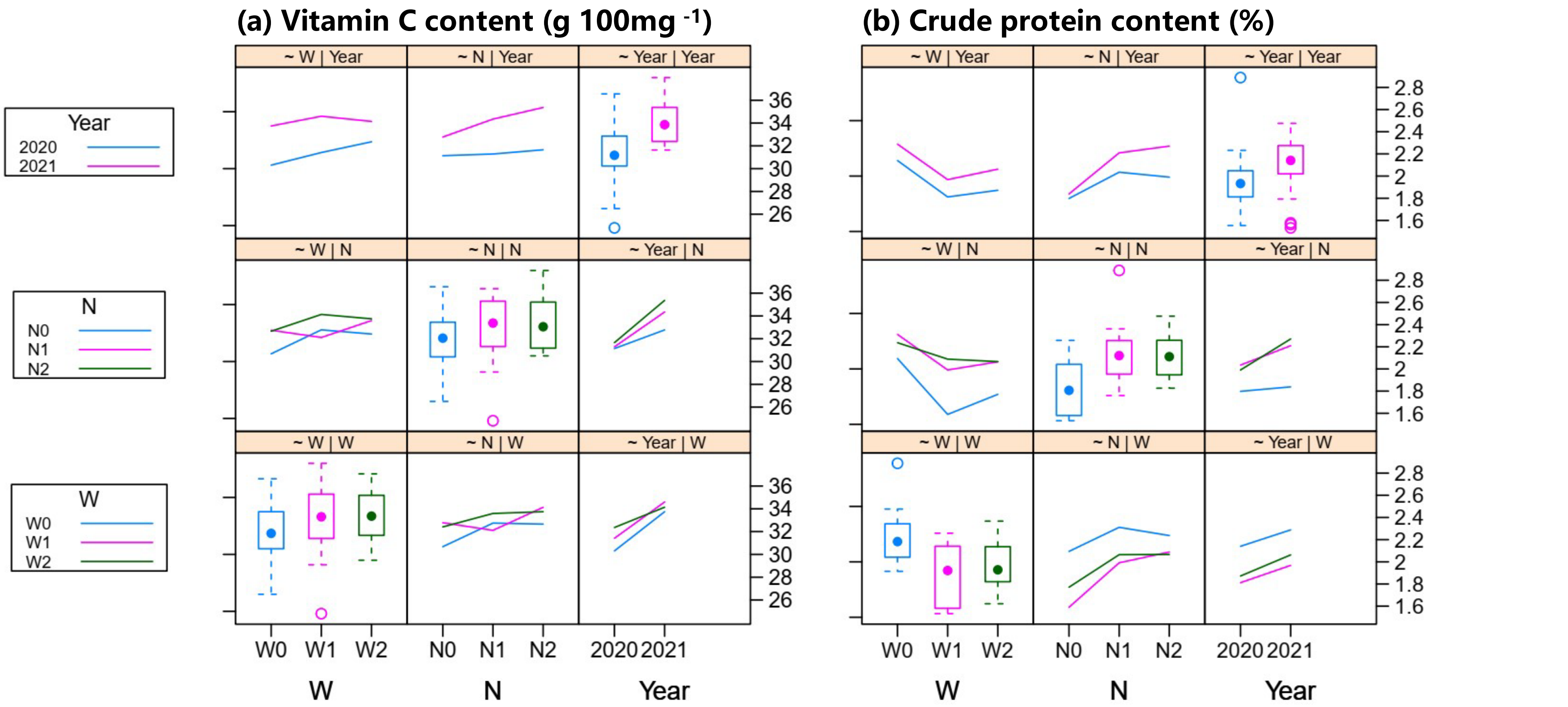

Figure S3. The main effect and 2-way interactions of water, nitrogen (N) treatments and years on the vitamin C and crude protein content of tubers. Water treatments: no water irrigation (W0), soil-based water irrigation regime (W1), and farmer's conventional water irrigation regime (W2). Nitrogen treatments: no N application (N0), 100% chemical fertilizer N at 210 kg N ha^{-1} application rate (N1), and 25% substitution of chemical N with manure N (N2).

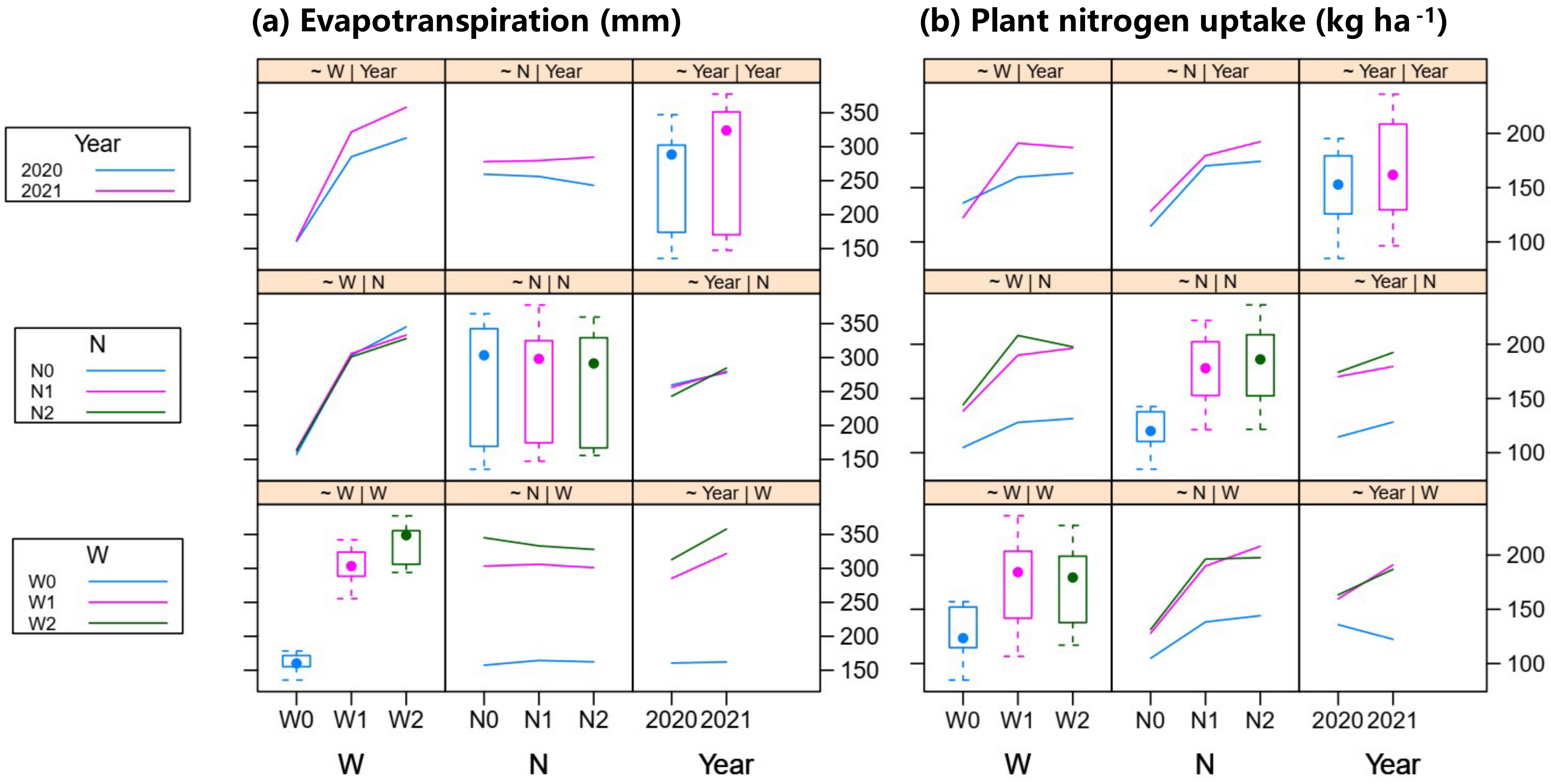

Figure S4. The main effect and 2-way interactions of water, nitrogen (N) treatments and years on the evapotranspiration and plant nitrogen uptake of potato. Water treatments: no water irrigation (W0), soil-based water irrigation regime (W1), and farmer's conventional water irrigation regime (W2). Nitrogen treatments: no N application (N0), 100% chemical fertilizer N at 210 kg N ha^{-1} application rate (N1), and 25% substitution of chemical N with manure N (N2).

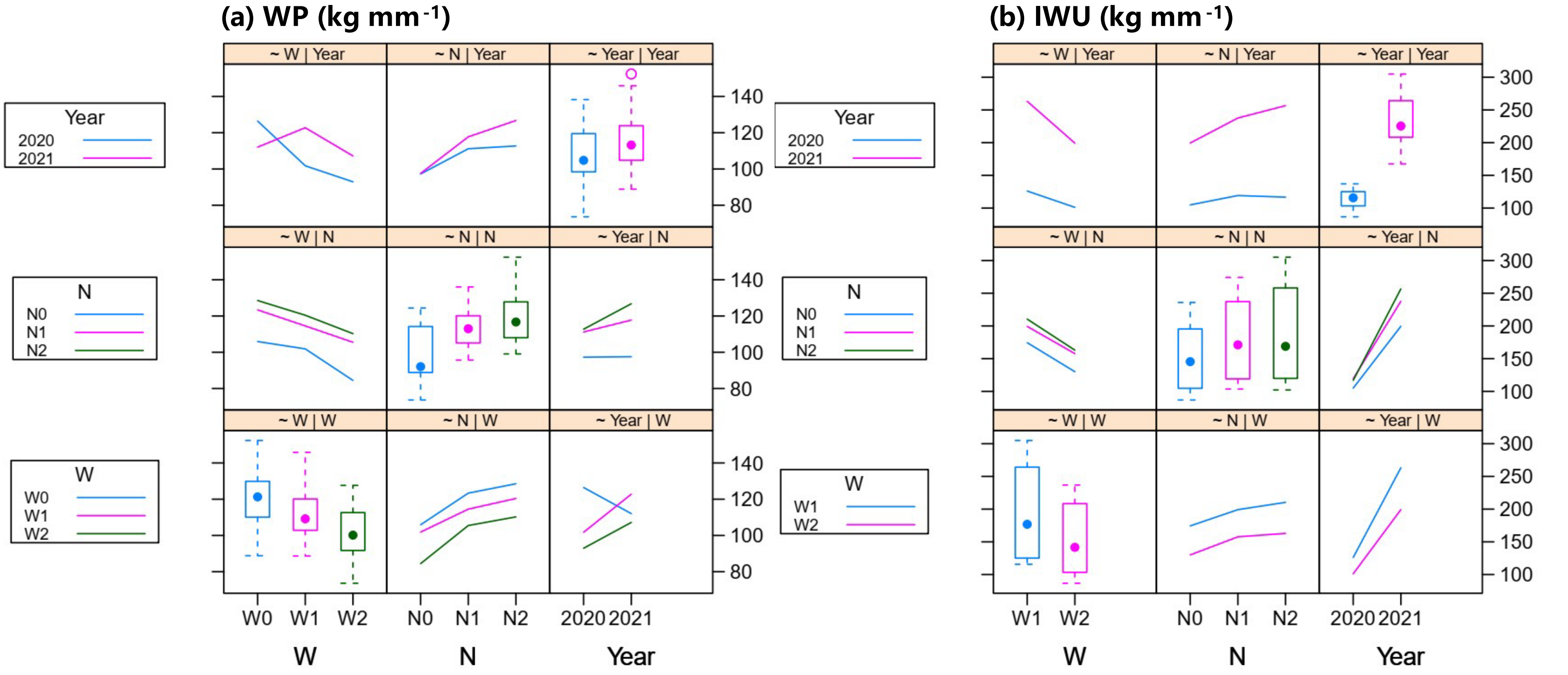

Figure S5. The main effect and 2-way interactions of water, nitrogen (N) treatments and years on the water productivity (WP) and irrigation water use efficiency (IWUE) of potato. Water treatments: no water irrigation (W0), soil-based water irrigation regime (W1), and farmer's conventional water irrigation regime (W2). Nitrogen treatments: no N application (N0), 100% chemical fertilizer N at 210 kg N ha^{-1} application rate (N1), and 25% substitution of chemical N with manure N (N2).

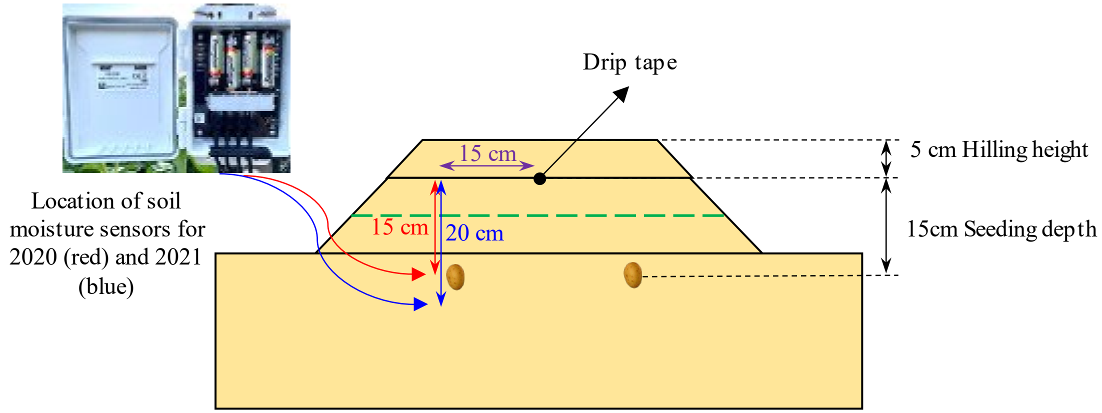

Figure S6. The cross-sectional view of the bed in Figure 1.

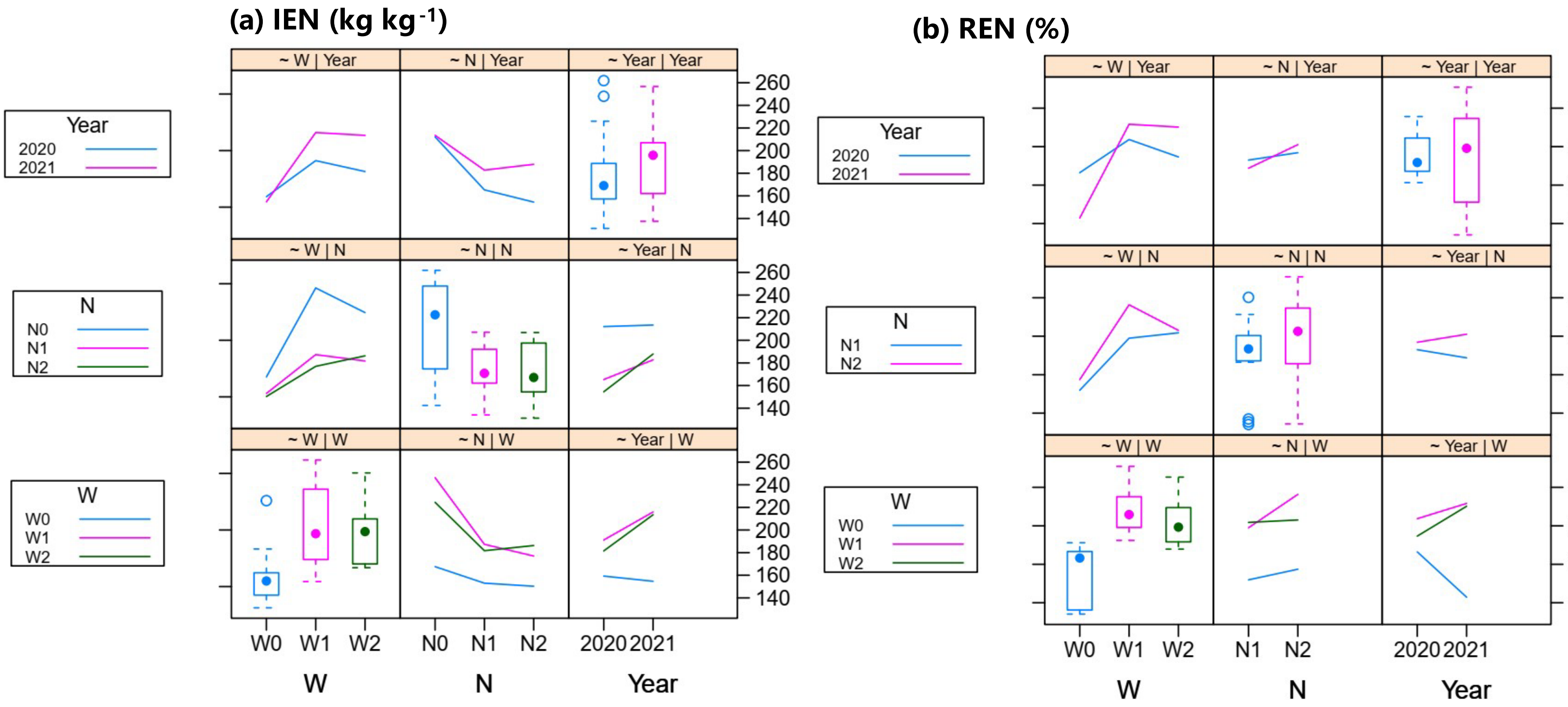

Figure S7. The main effect and 2-way interactions of water, nitrogen (N) treatments and years on internal efficiency of nitrogen (IEN) and recovery efficiency of nitrogen (REN) of potato. Water treatments: no water irrigation (W0), soil-based water irrigation regime (W1), and farmer's conventional water irrigation regime (W2). Nitrogen treatments: no N application (N0), 100% chemical fertilizer N at 210 kg N ha^{-1} application rate (N1), and 25% substitution of chemical N with manure N (N2).

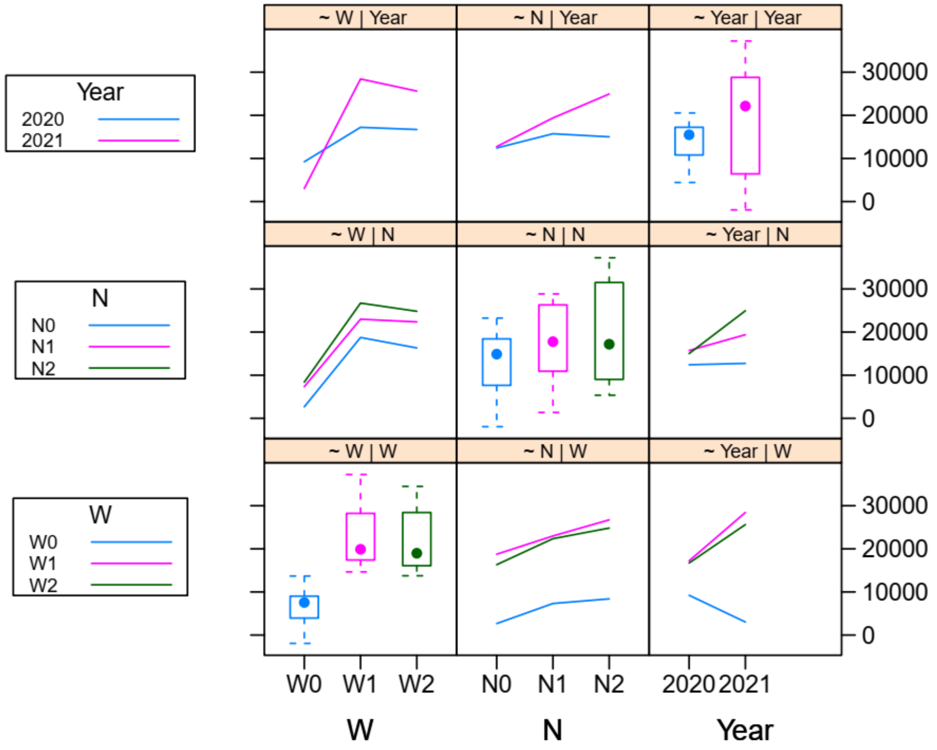

Figure S8. The main effect and 2-way interactions of water, nitrogen (N) treatments and years on net return. Water treatments: no water irrigation (W0), soil-based water irrigation regime (W1), and farmer's conventional water irrigation regime (W2). Nitrogen treatments: no N application (N0), 100% chemical fertilizer N at 210 kg N ha^{-1} application rate (N1), and 25% substitution of chemical N with manure N (N2).

Table S1. The leaf area index (LAI), tuber yield, commodity tuber ratio (CTR), vitamin C content (VC), crude protein content (CP), water productivity (WP), irrigation water use efficiency (IWUE), internal efficiency of nitrogen (IEN), recovery efficiency of nitrogen (REN) and net return under different water (W) and nitrogen (N) treatments in 2020 and 2021.

Year	Treatment [1]		LAI	Yield	CTR	VC	CP	WP	IWUE	IEN	REN	Net return
	W	N		(t ha^{-1})	(%)	(mg 100g^{-1})	(%)	(kg m^{-3})	(kg m^{-3})	(kg kg^{-1})	(%)	(CNY ha^{-1})
2020	W0	N0	3.7a [2]	18.1b	55.4b	28.7a	2.0a	12.1a	NA	186.1a	NA	6642.9b
		N1	5.0a	22.6a	66.4a	31.3a	2.3a	12.9a	NA	152.1ab	24.1a	11519.0a
		N2	5.5a	20.3ab	65.5a	30.9a	2.1a	12.9a	NA	139.5b	22.3a	9517.8ab
		Mean	4.7A	20.3B	62.4B	30.3A	2.1A	12.6A	NA	159.3B	23.2B	9226.6B
	W1	N0	3.6b	27.5b	66.2a	32.7a	1.6b	9.4b	12.0b	243.0a	NA	15598.6b
		N1	5.1a	30.4a	68.4a	29.7a	1.9a	10.6a	13.2a	175.2b	28.6b	18182.5a
		N2	6.2a	29.0ab	71.2a	31.8a	2.0a	10.6a	12.6ab	155.2b	35.1a	17857.3ab
		Mean	5.0A	29.0A	68.6A	31.4A	1.8B	10.2B	12.6A	191.1A	31.8A	17212.8A
	W2	N0	4.1b	25.7b	70.1a	32.0a	1.7a	7.7b	9.0b	207.2a	NA	14999.2a
		N1	6.2a	30.3a	66.4a	32.8a	2.0a	9.9a	10.6a	168.6b	26.8a	17460.5a
		N2	5.8a	30.7a	65.3a	32.3a	1.9a	10.3a	10.7a	168.8b	27.8a	17659.4a
		Mean	5.4A	28.9A	67.3AB	32.4A	1.9B	9.3B	10.1B	181.5AB	27.3B	16706.4A
	Mean	N0	3.8B	23.8A	63.9A	31.1A	1.8B	9.7A	10.5A	212.1A	NA	12413.6A
		N1	5.5A	27.7A	67.1A	31.3A	2.0A	11.1A	11.9A	165.3B	26.5A	15720.7A
		N2	5.8A	26.7A	67.3A	31.7A	2.0AB	11.3A	11.7A	154.5B	28.4A	15011.5A
2021	W0	N0	3.7b	15.0b	23.6c	32.6a	2.1b	9.0b	NA	149.1a	NA	-1327.5c
		N1	4.5a	18.1ab	46.9b	34.2a	2.3a	11.8ab	NA	153.9a	7.8a	3121.6b
		N2	4.7a	21.3a	54.5a	34.4a	2.4a	12.8a	NA	161.1a	15.1a	7264.2a
		Mean	4.3B	18.1B	41.7B	33.7A	2.3A	11.2AB	NA	154.7B	11.4B	3019.4B
	W1	N0	4.1c	34.3c	61.9c	32.8b	1.6b	11.0b	22.9c	249.6a	NA	21901.6c
		N1	5.6b	39.9b	67.2b	34.5ab	2.1a	12.3ab	26.6b	199.5b	30.4b	27790.4b
		N2	6.5a	44.2a	75.4a	36.5a	2.2a	13.5a	29.5a	198.8b	41.2a	35597.5a
		Mean	5.4A	39.5A	68.2A	34.6A	2.0B	12.3A	26.3A	216.0A	35.8A	28429.9A
	W2	N0	4.5b	32.8b	53.5b	32.8a	1.8b	9.2b	17.0b	241.8a	NA	17638.9b
		N1	6.8a	40.3a	65.1ab	34.4a	2.2a	11.2a	20.9a	194.8b	35.0a	27261.0a
		N2	7.3a	42.1a	72.4a	35.2a	2.2a	11.8a	21.9a	203.8b	35.2a	31976.5a
		Mean	6.2A	38.4A	63.7A	34.1A	2.1AB	10.7B	19.9B	213.5A	35.1A	25625.5A
	Mean	N0	4.1B	27.3A	46.3B	32.8B	1.8B	9.8B	19.9B	213.5A	NA	12737.7B
		N1	5.6A	32.8A	59.7A	34.3A	2.2A	11.8A	23.8AB	182.7A	24.4A	19391.0AB
		N2	6.2A	35.9A	67.5A	35.4A	2.3A	12.7A	25.7A	187.9A	30.5A	24946.1A

[1] Water levels: no water irrigation (W0), soil-based water irrigation regime (W1), and farmer's conventional water irrigation regime (W2). Nitrogen levels: no N application (N0), 100% chemical fertilizer N at 210 kg N ha^{-1} application rate (N1), and 25% substitution of chemical N with manure N (N2).

[2] Lowercase letters indicate differences between treatments. Uppercase letters indicate differences between water or nitrogen levels. Different letters in the same column for each year indicate significant differences ($P < 0.05$). The values with NA are missing values.

Table S2. The net return (CNY ha^{-1}) under different experimental years.

Year	Tuber income	Fertilizer cost	Seed cost	Pesticide cost	Labor cost	Irrigation cost	Net return
2020	23055	2244	6000	0	4500	429	14382
2021	30108	2897	7000	900	6000	285	19025
